# Gross Deception

**Published:** 1844-09-07

**Authors:** 


### THE MEDICAL EXAMINER,

### And Record of Medical Science.

Vol. VII.] PHILADELPHIA, SATURDAY, SEPTEMBER 7, 1844. [No. 18.

### GROSS DECEPTION.

#### NEW YORK REPRINT OF THE LONDON LANCET.

MR. EDITOR,—The London "Lancet" is *said* to be reprinted in New York, by which would, of course, be understood the *whole* of the Lancet. Perhaps it might be considered justifiable on the part of the publishers to exclude professional matters of mere local interest, which are not so important to us on this side the Atlantic; but surely they who subscribe for the New York edition have a right to expect, that every thing of general professional interest is furnished them. To enable the subscribers to judge on this matter, and to understand fully the difference between the two "Lancets," we give in *parallel columns*, the contents of No. 1087 of "the Lancet" for June 29, 1844; and alongside of them those of the "London Lancet" of New York, marked "London Ed., No. 1087, Am. Ed., No. 85;" distinguishing in *Italics* those articles of the London edition that have been omitted in the reprint.

| LANCET. | NEW YORK REPRINT. |
|---|---|
| *Lectures on the operations of Surgery, and on Diseases and accidents requiring operations, by Robert Liston, Esq. (Lecture III.)* | |
| Observations on Organic Chemistry and its relations to Physiology, by Justus Liebig, M. D. Ph. D. (concluded.) | Observations on Organic Chemistry, and its relations to Physiology, by Justus Liebig, M. D. Ph. D. (concluded.) |
| *On the Detection of Poisons generally, and on a new method for the detection of Arsenic, by Dr. Remigius Fresenius. Giessen* (Part 3.) | |
| On Poisonous Drugs; the danger of their indiscriminate use, and their remedies, by Sir Geo. Lefevre, M. D. | On Poisonous Drugs; the danger of their indiscriminate use, and their remedies, by Sir Geo. Lefevre, M. D. |
| *Practical Facts and Observations on Diseases of women, and some subjects connected with the Practice of Midwifery, by G. O. Heming, M. D.* | |
| On the treatment of Lepra vulgaris, by Dr. John Charles Hall, East Retford. | On the treatment of Lepra vulgaris, by Dr. John Charles Hall, East Retford. |
| On the use of Alkalies in Rheumatism. By Dr. J. J. Furnivall. | On the use of Alkalies in Rheumatism. By Dr. J. J. Furnivall. |
| Dr. Wigan on Duality of the Mind. | Dr. Wigan on Duality of the Mind. |
| FOREIGN DEPARTMENT. | FOREIGN DEPARTMENT. |
| The religious mania which reigned in Sweden in 1841-42. Loss of Speech from Lesion of the Brain. On the use of large doses of Nitrate of Potass. | The religious mania which reigned in Sweden in 1841-42. Loss of Speech from Lesion of the Brain. On the use of large doses of the Nitrate of Potass. |
| BRITISH MEDICAL JOURNALS. | BRITISH MEDICAL JOURNALS. |
| On the Functions of the true spinal system. Acceleration of puberty in the female by factory labor. On the signs of actual death. | On the Functions of the true spinal system. Acceleration of puberty in the female by factory labour. On the signs of actual death. |
| CHEMISTRY, PHARMACY AND MATERIA MEDICA. | CHEMISTRY, PHARMACY AND MATERIA MEDICA. |
| On the Hyoscyamus niger. | On the Hyoscyamus niger. |
| *The Medical Bill again postponed.* | |
| *Organization of the Medical profession in Prussia. The different classes of Practitioners.* | |
| HOSPITAL REPORTS. | HOSPITAL REPORTS. |
| *Royal London Ophthalmic Hospital—rheumatic ophthalmitis.* | ST. GEORGE'S HOSPITAL. Paralysis from pressure on the spinal cord. |
| *Royal College of Surgeons in Ireland.* | LONDON HOSPITAL, Hæmorrhagic Diathesis. |
| *Charter of the College of Surgeons and the younger members of the College.* | MEDICAL SOCIETIES. |
| *Anatomy in Edinburgh.* | ROYAL MEDICAL and CHIRURGICAL SOCIETY. Cases of Tubular Expectoration. |
| *Joshua Brookes's method of Preserving Bodies for Dissection.* | |
| *Medical Protection Assembly.* | |
| *News of the Week.* | |
| *Mortality Table.* | |
| *College of Surgeons.* | |
| *Apothecaries' Hall.* | |
| *Correspondents.* | |

It thus appears, that the valuable Lectures of Liston, occupying 8½ of the large columns of the London work, and illustrated with two wood-cuts; the communication of Fresenius, occupying 3½ columns; and that of Dr. Heming, occupying 5½ columns—both on important subjects—are not contained in the New York edition; and if we add the space occupied by the other articles omitted, amounting to 11 columns, we have, on the whole, 28 columns omitted of 38—the whole reading contents of No. 1087 of the London Lancet!

It is proper to add, that in the New York No. 1087, six of its comparatively small columns are occupied with HOSPITAL REPORTS and TRANSACTIONS of MEDICAL SOCIETIES not one of which is to be found in the number of which it professes to be a reprint.

Surely some explanation is due to the subscribers and the profession for such unexampled deception; as well as for the additional outrage of advertising on the cover of a professional Journal, the trumpery and indecent production entitled "Becklard's Physiological Mysteries and Revelations in Love, Courtship and Marriage," as "a highly important work!"

SCRUTATOR.

*Philadelphia*, Aug. 29, 1844.

We have examined the two "Lancets," and regret to find, that the account of our correspondent is but too true.—ED.

